# Solar forcing on elemental and nanomechanical variations in Late Cretaceous lacustrine deposits

**DOI:** 10.1038/s41598-025-27521-9

**Published:** 2025-12-21

**Authors:** Yuke Liu, Xiaomei Wang, Kunning Cui, Minghao Wu, Xuening Qi, Shuichang Zhang

**Affiliations:** 1 Research Institute of Petroleum Exploration and Development, Beijing, 100083 China; 2 State Key Laboratory of Continental Shale Oil, Daqing, 163712 China; 3 PetroChina Daqing Oilfield Co., Ltd, Daqing, 163712 China

**Keywords:** Nanomechanical behavior, Lacustrine deposits, Solar activities, Decennial to annual cycles, Climate sciences, Solid Earth sciences

## Abstract

Decennial to annual-scale paleoclimate proxy variations are often attributed to solar forcing and/or unforced internal climate oscillations, but recognition of such variations in nanomechanical properties is extremely rare. Here we present a well-preserved, millimeter-scale laminated shale-carbonate couplet from Late Cretaceous petroliferous Qingshankou Formation in the Songliao Basin, NE China (ca. 90.53 Ma). Cyclicity analyses of elemental and nanomechanical data reveal periods of 47.7 ± 5.2–20.3 ± 2.2 year and 15.1 ± 1.6–7.4 ± 0.8 year attributed to Hale and Schwabe solar cycles, respectively; a 5.3 ± 0.6–2.5 ± 0.3 year period probably reflecting ENSO-like climatic fluctuations. Solar activities govern climatic changes that reshape aquatic environment, which further controls the laminated sedimentary composition and mechanical properties. The mechanically weak interfaces between solar-forced biogenic soft carbonates and terrigenous hard detritus are susceptible to crack propagation, affecting horizontal hydraulic fracturing in shale oil exploitation. This study introduces an integrated science-engineering research paradigm in shale oil field.

**Supplementary Information:**

The online version contains supplementary material available at 10.1038/s41598-025-27521-9.

## Introduction

Since the onset of shale-oil revolution in lacustrine basins, substantial research has focused on understanding shales, especially those laminated ones, as both prolific source rocks and oil reservoirs^1,2^. Laminae in shales are featured by different lithofacies with various oil generation potential, reserving capacity and mechanical property, leading to the inconsistency between geological and fracturing “sweet spots” ^3^. The mechanically weak interfaces in laminated shales with high-density beddings could lead to complex fracture morphology and restrict hydraulic fracturing from sufficient lengths and heights, being a main restraint on effective shale oil exploration^4^, whereas the environmental driving force remains unclear.

The mechanism of chemical laminations in shale has been clarified. Centennial to interannual scale elemental cyclicities had been identified in lacustrine laminated shales from the Proterozoic to Cenozoic, e.g., Torridonian (Mesoproterozoic) and Middle Devonian strata in Scotland^5^, Triassic Yanchang Formation in Ordos Basin, NW China^6^, Early Cretaceous Shinekhudag Formation in SE Mongolia^7^, Early Cretaceous Yixian Formation in western Liaoning, NE China^8^, Late Cretaceous Nenjiang Formation in Songliao Basin, NE China^9^, Eocene Green River Formation in Uinta Basin^10^, Eocene Shahejie Formation in Bohai Bay Basin, NE China^11^ and Late Tertiary Gray Fossil Site in NE Tennessee^12^, which are interpreted to reflect solar cycles (i.e., Schwabe ~ 11 year, Hale ~ 22 year, Gleissberg 80–100 year, de Vries-Suess 200–250 year)^13,14^ and/or internal climate system variations (i.e., ENSO 3–7 year)^15^.

However, the driving force for mechanical laminations remains understudied, with priority given to mechanical characterization^16,17^ and upscaling model^18,19^. The elemental content (or mineralogical constituent) is the primary factor influencing mechanical properties of shale^20^. Correlation between mechanical properties and elemental (mineralogical) contents has been investigated from micrometer^21^, centimeter^22^ to meter^20^ scales. Due to the inherent heterogeneity of shale, both elemental compositions and mechanical properties exhibit highly non-linear and unstable characteristics. Traditional linear and nonlinear regression methods could only reveal the variation characteristics between elements and mechanics on a single time scale, thereby impossible to probe into the evolution relationship and interaction characteristics on multiple time scales. The coupling of spectral and wavelet analyses has been widely applied in the field of cyclostratigraphy and hydrometeorology^23^, whereas little trial has been made on geomechanics.

This study for the first time reports elemental and nanomechanical cyclicity results of a laminated shale from Late Cretaceous petroliferous Qingshankou Formation (K_2_qn) in the Songliao Basin, NE China^24,25^. Environmental factors, including external solar activity forcing and internal climate oscillations, had been assessed in determining elemental and mechanical variations in prolific hydrocarbon source rocks. This work provides a novel science-engineering integrated framework, which has significance in hydraulic fracturing design for shale oil exploitation.

## Geological setting and sampling

**Fig. 1:**
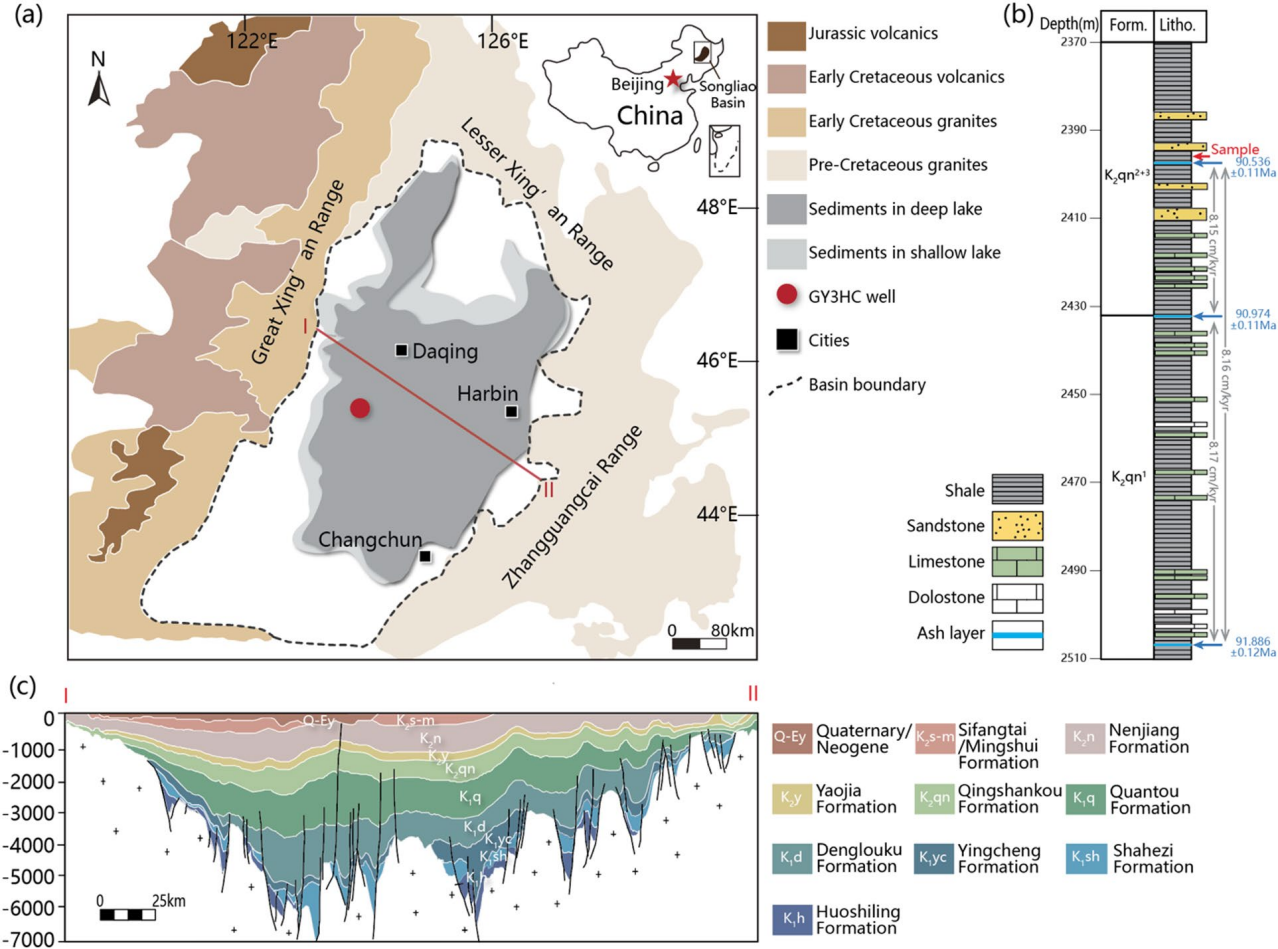
(**a**) Modern schematic map of the Songliao Basin and (**b**) the lithology of GY3HC well. The structural cross section (I-II) along the central part of the Songliao Basin in (**a**) is shown in (**c**). Red arrow in (**b**) points to sample position. Blue arrows in (**b**) mark ash layers with zircon U-Pb ages referred from^29^. Sedimentation rates are calculated using U-Pb ages of ash layers. (**a,c**) are modified from^30^.

The Songliao Basin in NE China (Fig. 1a) contains ~ 7,000 m of Cretaceous-Cenozoic lacustrine sediments (Fig. 1c)^26,27^. The location of the basin remains basically unchanged as the paleolatitude at Cretaceous time (40–50°N) was quite similar to now (42.5–49.5°N) ^28^. The GY3HC well at the depocenter (Fig. 1a) preserves ~ 140 m thick strata of the K_2_qn, including black shales with interbedded carbonates and sandstones (Fig. 1b). Three volcanic ash layers observed in this well at depth of 2507.0 m, 2432.5 m, 2397.6 m serve as chronostratigraphic anchors for stratigraphic correlation^25^, and their ages can be referred from adjacent SK-1s well with precise U-Pb zircon ages of 91.886 ± 0.12, 90.974 ± 0.11, and 90.536 ± 0.11 Ma, respectively^29^ (Fig. 1b). The lower part of Member 2 + 3 in Qingshankou Formation (K_2_qn^2 + 3^) is the targeted strata for shale oil exploration by hydraulic fracturing.

## Methodology

### Sample preparation

A mudstone with carbonate laminae was selected from a depth of 2396.6 m in GY3HC well (Fig. 1b). The thin section with a length of ~ 2 cm was made, which falls within the range of previous studies (1.5–25 cm) about solar forcing on lacustrine rhythmites^5,6,8,10–12^. The surface of thin section was mechanically polished before elemental and nanomechanical tests. Silicon carbide sandpaper with sizes from 1200 to 2000 grit was firstly used. Then a polishing machine with aluminum oxide suspension polishing fluid (grain sizes of 0.25 μm) was used for 30 min to guarantee a smooth surface. Additionally, acknowledgment is made that extrapolating insights from a single thin section to the entire basin may have limitations, but the first trial is still instructive.

### Elemental and principal component analyses

**Fig. 2:**
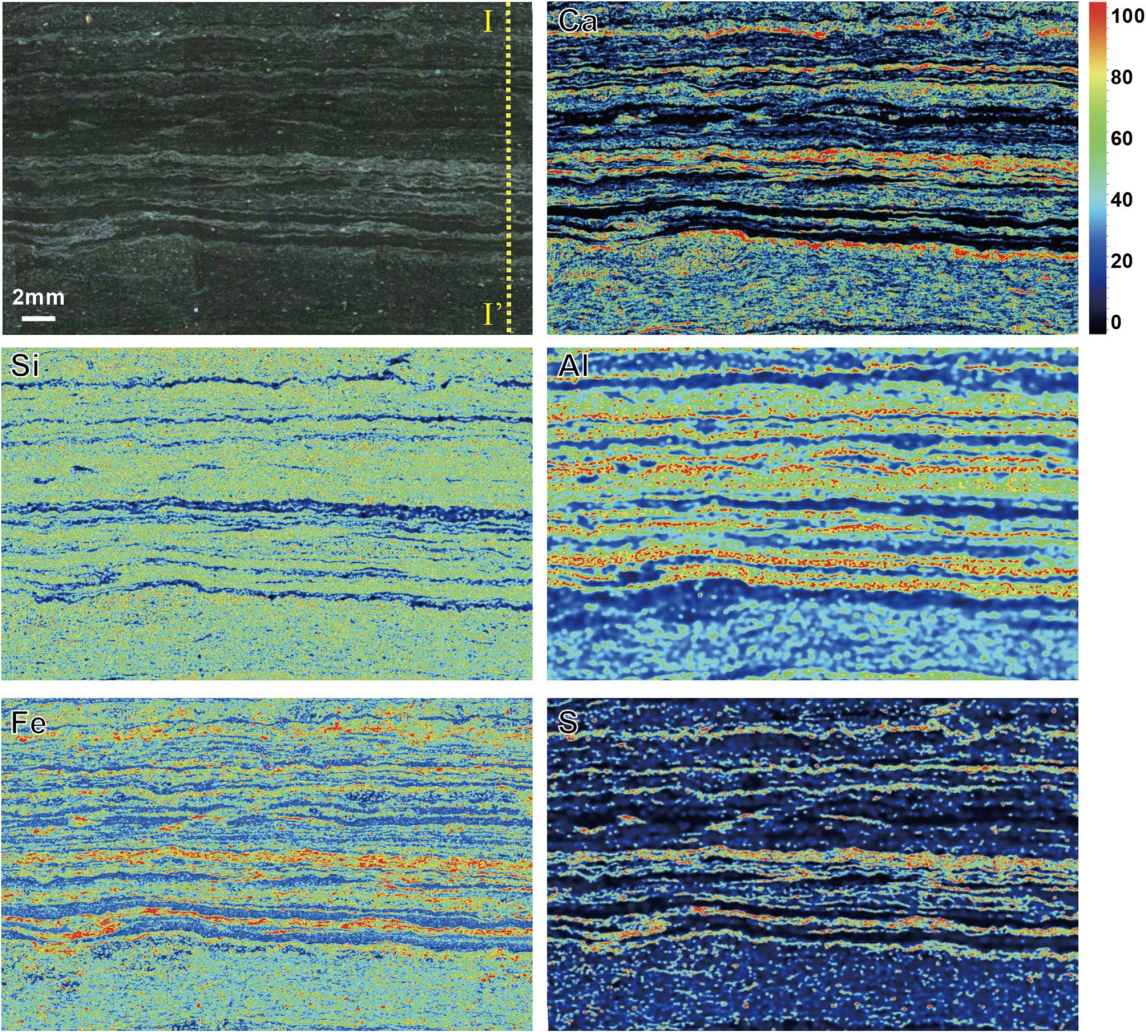
Photograph and multi-elemental (Ca, Si, Al, Fe and S) imaging of the sample by using XRF (27 μm pixel size; 4 ms exposure time per pixel). All images share the same color bar.

Elemental mapping of the sample section (Fig. 2) was obtained via a M4 Tornado X-ray Fluorescence (Bruker Company, Germany) at Research Institute of Petroleum Exploration and Development, Beijing, China. The sample was scanned with a 20-µm-diameter X-ray beam and a single-point exposure time of 4 ms. Elemental images of Ca, Si, Al, Fe and S were obtained with a 27 μm-spatial resolution.

Elemental line scanning along the yellow dashed line in Fig. 2 was conducted on a RESOlution 193 nm LA (Australian Scientific Instruments, Australia) with a PlasmaQuant MS Elite ICP-MS (Analytik Jena AG Company, Germany) at Beijing Createch Testing Technology Co., Ltd., Beijing, China. The spot size and scanning rate were set as 10 μm and 50 μm/s, respectively. A total of 750 points with an interval of ~ 27 μm was obtained. The Data was analyzed via software Iolite 3.25 ^31^. Elemental distributions of Si, Al, K, Mg, Ca, Mn, Fe and S were selected for principal component analysis (PCA, Origin 8.0 software).

### Nanomechanical analysis

Nanoindentation was performed on an Anton Paar NHT^3^ with a diamond Berkovich indenter. The patented active surface referencing system in NHT^3^ and a constant room temperature could reduce thermal drift. The nanomechanical properties, including Young’s modulus and hardness, along the yellow dashed line in Fig. 2 were obtained by conducting 200 indentation tests with a spacing of 100 μm under the maximum indentation forces (*F*_*max*_) of 200 mN and 50 mN, respectively (indentation into the bedding plane). And these two loads resulted in an indent imprint with a depth of ~ 4 μm (diameter of ~ 20 μm) and ~ 1 μm (diameter of ~ 4 μm) on sample surface, respectively. Given the major grain size of this sample to be ~ 10 μm, the result would be more homogenous at *F*_*max*_=200 mN, while information of individual phase could be derived at *F*_*max*_=50 mN.

A fused silica standard specimen was used for tip shape imperfection calibration. For all tests on shale sample, continuous stiffness measurement was adopted during loading at a constant indentation strain rate of 0.1 s^− 1^ to avoid thermal drift^32^. After reaching *F*_*max*_, the load remained constant for 2s before linearly unloaded to 0 within 30 s, aimed at a short period to reduce potential thermal drift^33^. Young’s modulus (*E*) and hardness (*H*) are calculated as follows^34^:

1 $$\:\frac{1}{{E}_{r}}=\frac{1-{v}^{2}}{E}+\frac{1-{v}_{i}^{2}}{{E}_{i}}$$

where *v* is the Poisson’s ratio of sample (*v* = 0.3 is applied, as a range of 0.05–0.3 for Poisson’s ratio only leads to 8% variation in Young’s modulus^35^, *v*_*i*_ and *E*_*i*_ represent elastic indentation modulus and Poisson’s ratio of Berkovich indenter, respectively (*v*_*i*_ =0.07, *E*_*i*_ =1141 GPa). *Er* is the reduced modulus and calculated as:

2 $$\:{E}_{r}=\frac{\sqrt{\pi\:}S}{2\sqrt{{A}_{c}}}$$

where *S* is the contact stiffness, defined as the initial slope of the unloading force-displacement curve (*S* = *dF*/*dh*, *F* and *h* are force and depth, respectively). *A*_*C*_ is the contact area, calibrated by independent measurements on fused silica standard specimen by using area function (*A*_*C*_ = *C*_*0*_*h*_*C*_^*2*^+ *C*_*1*_*h*_*C*_+ *C*_*2*_*h*_*C*_^*1/2*^+ *C*_*3*_*h*_*C*_^*1/4*^+…+ *C*_*8*_*h*_*C*_^*1/128*^, where *C*_*0*_…*C*_*8*_ are constants determined by curve-fitting, *h*_*C*_ is contact depth)^32^. Considering sink-in effect, *h*_*C*_ is calculated as ^32^:

3 $$\:{{h}}_{{c}}={{h}}_{{m}{a}{x}}-0.75\frac{{{F}}_{{m}{a}{x}}}{{S}}$$

where *h*_*max*_ is the maximum indentation depth. Pile-up effect is negligible as most indents are featured by *h*_*f*_/*h*_*max*_ < 0.7 (*h*_*f*_ is the permanent depth after unloading)^32^.

Hardness can be obtained as:

4 $$\:H=\frac{{F}_{max}}{{A}_{c}}$$

### Cyclicity analysis

Cyclicity analysis (including spectral and wavelet analyses) of geochemical (including Ca, Al and S) and nanomechanical (Young’s modulus and hardness obtained at both 200 mN and 50 mN) series were performed using the Acycle v2.4.1 software^36^.

### Data pretreatment

Sampling intervals are 27 μm and 100 μm for elemental and nanomechanical data, respectively. As uniformly sampled series are required for power spectral analysis (multitaper method, MTM)^37^, all raw data series were linearly interpolated, and the spectral results before and after interpolation show little difference. Removal of long-term trends is a key step for power spectral analysis, to guarantee data variability oscillating around a zero mean, and to avoid power leakage from very low-frequency components into higher frequencies. In this study, all the series were 35% detrended via LOWESS (locally weighted scatterplot smoothing) method to remove long-term interfering.

### Spectral analysis

Spectral analysis can reveal the scale-dependent relationships between the variables but are only applicable to stationary systems. In this study, 2π-MTM with a classical red-noise null mode (widely used in millimeter scale geological samples^6,11^ is implemented and were reported at 85%, 90%, 95% and 99% confidence levels of spectral peak significance. Evolutionary spectral analysis as evolutionary fast fourier transform (EFFT) analysis was implemented for all series using a 3.9 mm sliding window and step of 0.03 mm to extract local-frequency information. Besides, Gaussian filter method was applied to isolate desired signals within a specified frequency range, including bandpass of 0.28 ± 0.08 cycles/mm and 1.0 ± 0.05 cycles/mm for Al series, 1.2 ± 0.1 cycles/mm for modulus at 200 mN, 1.6 ± 0.1 cycles/mm for hardness at 200 mN, 1.0 ± 0.04 cycles/mm for modulus at 50 mN, and 0.96 ± 0.04 cycles/mm for hardness at 50mN.

### Wavelet analysis

Wavelet analysis outperforms in analyzing time series containing nonstationary power at various frequencies^38^, including continuous wavelet transform (CWT) for localized intermittent oscillations in time series, cross-wavelet transform (XWT) and wavelet transform coherence (WTC) for the connection in time and frequency between two series. Elemental and nanomechanical series were subjected to wavelet analysis using ‘Morlet’ as mother wavelet.

In this study, the MTM power spectral analysis, EFFT and CWT were utilized to analyze the information of geochemical and nanomechanical series. XWT and WTC were applied to analyze the multi-temporal correlations between elemental contents and nanomechanical properties.

## Results

### Elemental distributions and PCA results

**Fig. 3:**
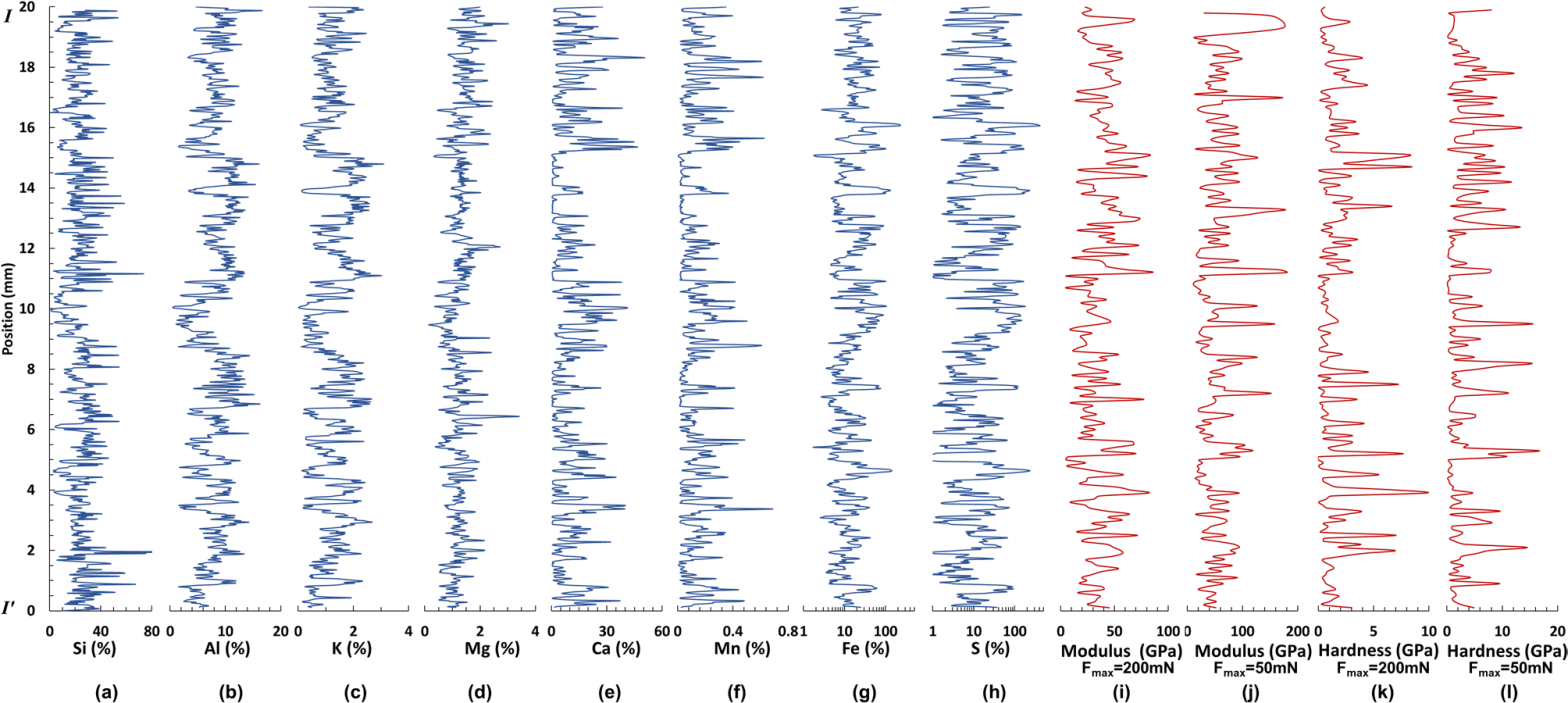
Elemental contents and nanomechanical properties along the yellow dashed line I-I’ in Fig. 2. Si (**a**), Al (**b**), K (**c**), Mg (**d**), Ca (**e**), Mn (**f**), Fe (**g**), S (**h**), Young’s modulus at 200 mN (**i**) and 50 mN (**j**), hardness at 200 mN (**k**) and 50 mN (**l**).

The sample preserves millimeter-scale alternations between white carbonate laminae (termed “light”) and dark detritus laminae (termed “dark”) (Fig. 2). The elemental images are shown in Fig. 2. Quantitative elemental variations along the yellow dashed line in Fig. 2 are plotted in Figs. 3a-h. The light laminae are rich in Ca, Mn, Fe and S, whereas depleted in Si, Al and K compared to the dark laminae (Fig. 2).

**Fig. 4:**
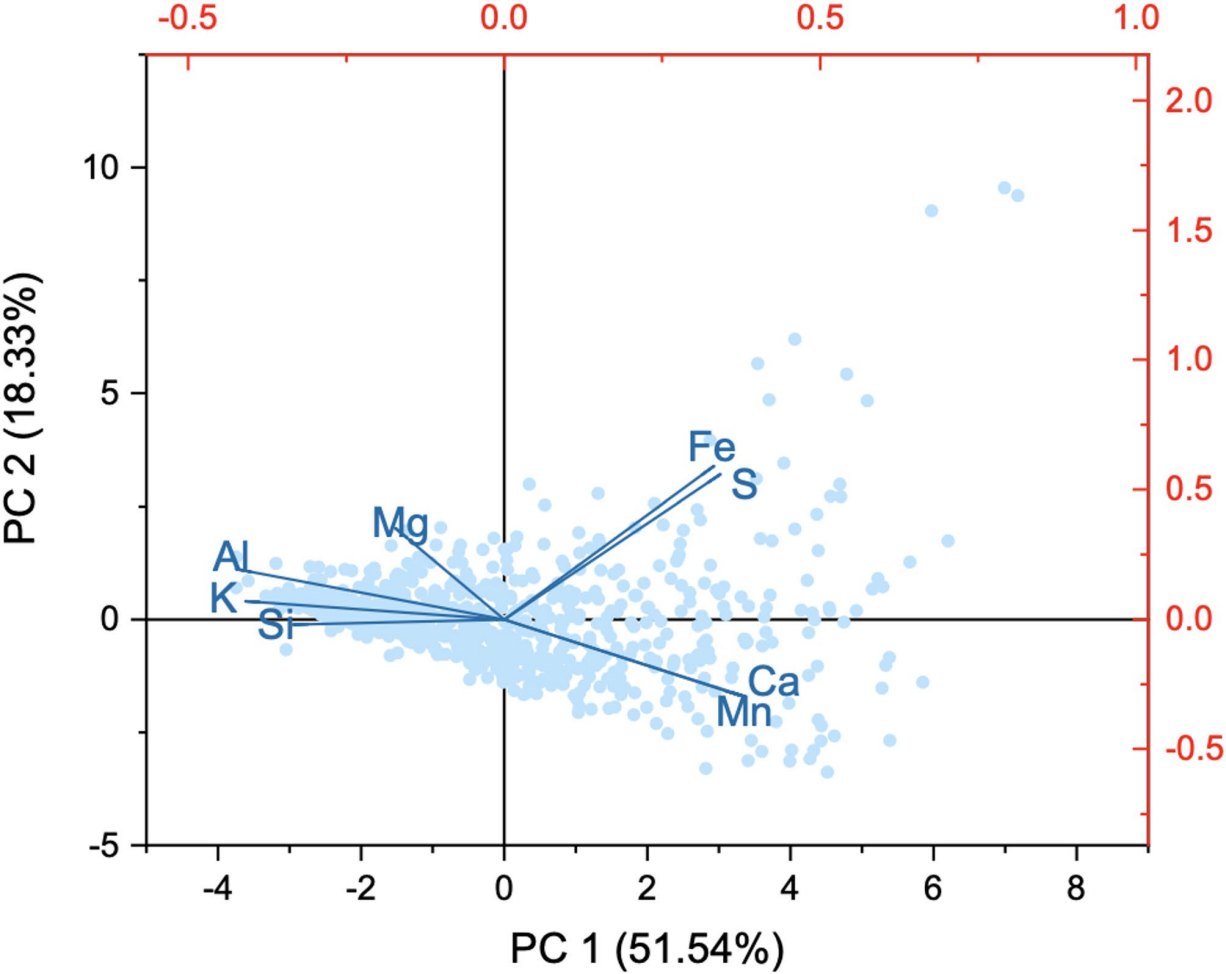
Sample (blue dots) and elemental loading (red lines) biplot of PC1 versus PC2 for elements.

According to PCA of elemental distributions in Fig. 4, the first three principal components account for 51.54% (PC1),18.33% (PC2) and 12.21% (PC3), i.e., a total of 82.19% of the total variance (Table S1). PC1 is interpreted as endogenic signal with negative Si, Al, K and Mg loadings but positive Ca, Mn, S and Fe loadings (Fig. 4). In the PC2 axis, Ca, Mn, S and Fe are subdivided, as Fe and S (pyrite) have positive loading values, and Ca and Mn (carbonate) have negative loading values (Fig. 4). Strong positive correlations are observed between Al and K, Ca and Mn, Fe and S; while four pairs, including Al vs. Ca, Al vs. Mn, K vs. Ca, and K vs. Mn, show strong negative relationship (Table S2). In this view, three groups are identified (Table S1), and Al, S and Ca are selected as representative elements for further cyclicity analysis.

### Nanomechanical variations

**Fig. 5:**
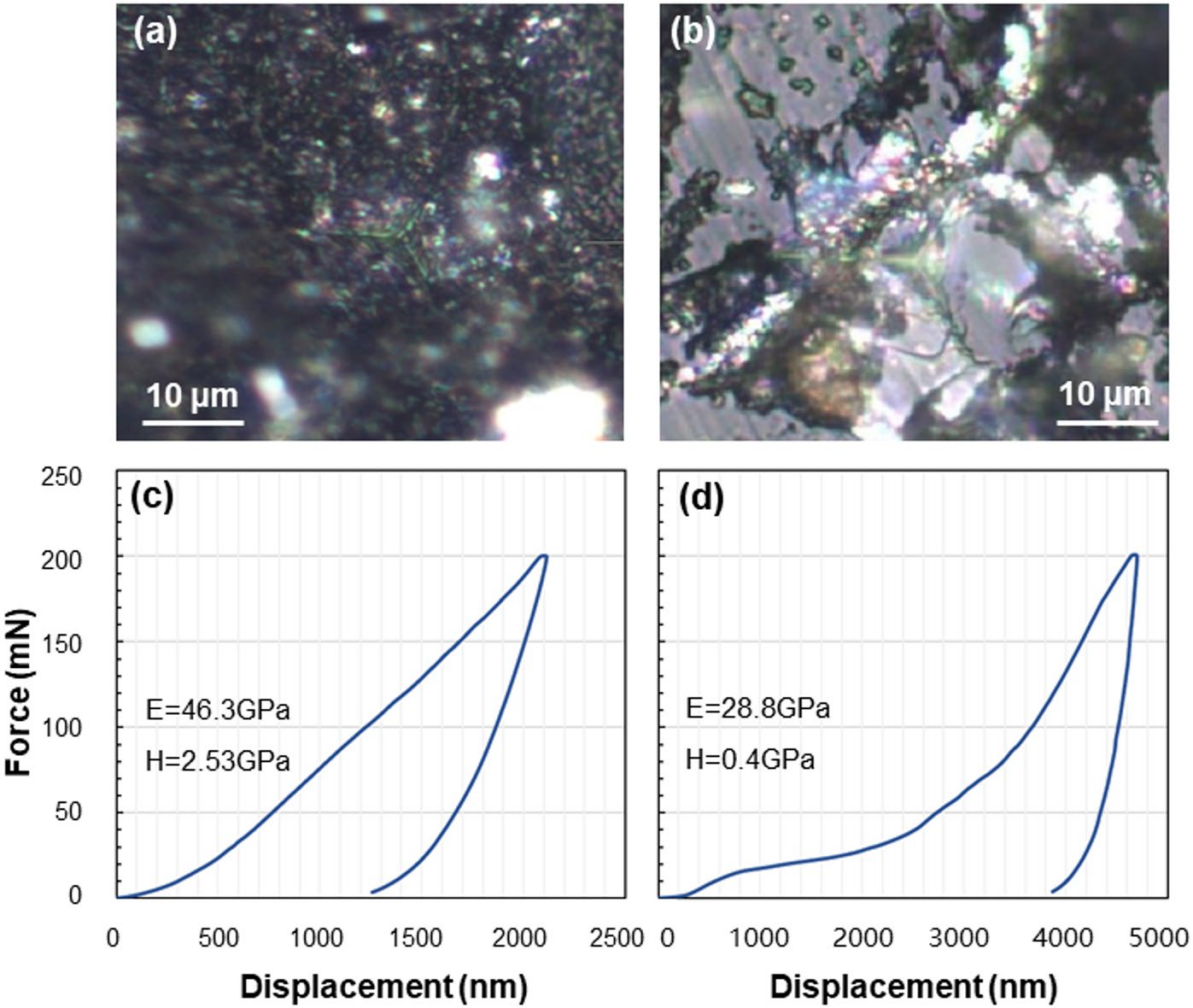
Representative indents and force curves on terrigenous detritus (**a,c**) and calcareous particles (**b,d**) in shale at *F*_*max*_=200 mN. Values of modulus (E) and hardness (H) are shown in (**c,d**).

Young’s modulus and hardness obtained at *F*_*max*_=200 mN and *F*_*max*_=50 mN along the yellow dashed line in Fig. 2 are shown in Fig. 3i-l. Representative indents left on different phases in shale at *F*_*max*_=200 mN are shown in Fig. 5a, b. The collapse of brittle minerals led to a relatively larger indent with weaker mechanical property (E = 28.8 GPa, H = 0.4 GPa) (Fig. 5d) compared to terrigenous clay (E = 46.3 GPa, H = 2.5 GPa) (Fig. 5c). According to the histogram (Figs.S1 a and b) and boxplot (Figs.S1 c and d) of modulus and hardness, the values are less with narrower distributions under *F*_*max*_=200 mN when compared to *F*_*max*_=50 mN, as a result of indentation size effect^39,40^.

### Cyclicity results

#### Spectral analysis

The sampling intervals for elemental and nanomechanical data are 27 μm and 100 μm, respectively. According to the guideline that sampling frequency should be greater than twice (four times even better) the frequency of the highest-frequency signal analyzed^41^, only periods higher than 0.1 mm and 0.4 mm are considered reliable for elemental and nanomechanical series, respectively. And only cycles above 0.4 mm are reliable for cross-proxy comparison.

**Fig. 6:**
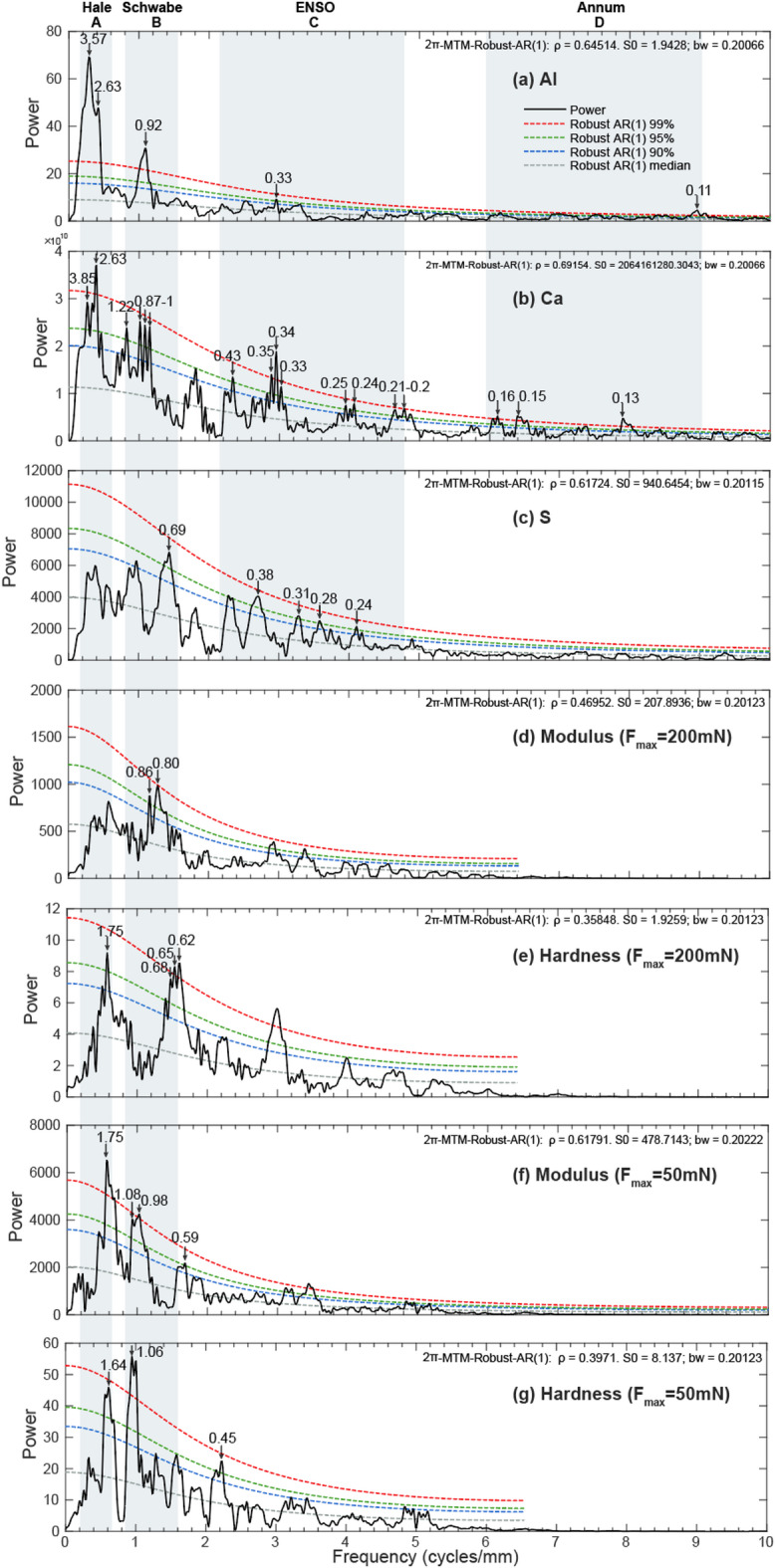
2π MTM power spectra of elements Al (**a**), Ca (**b**), S (**c**), and Young’s modulus and hardness at 200 mN and 50 mN (d-g) with the first-order autoregressive (AR(1)) red noise model of median, 90%, 95%, and 99% confidence levels. Black arrows mark the peaks with > 95% confidence levels while higher than 0.1 mm for elemental series and 0.4 mm for nanomechanical series with units of mm. Major cycle bands are shaded in gray.

MTM power spectral analyses of detrended Al, Ca and S series show hierarchies of significant spectral peaks at > 95% confidence levels (Figs. 6a-c). Al series shows sharp peaks at 3.57 mm, 2.63 mm, 0.92 mm, 0.11 mm (> 99% confidence levels) and 0.33 mm (> 95% confidence levels) wavelengths (Fig. 6a). Ca series reveals a hierarchy of cycles with 2.63 mm, 0.34 mm, 0.16–0.15 mm wavelengths with over 99% confidence; and 3.85 mm, 1.22 mm, 1–0.87 mm, 0.24–0.25 mm, 0.2–0.21 mm above 95% confidence level (Fig. 6b). S data series has no peaks exceeding 99% significance level, while expressing cycles at 0.69 mm, 0.38 mm, 0.31 mm, 0.28 mm and 0.24 mm with over 95% confidence (Fig. 6c).

As for nanomechanical series, the detrended Young’s modulus at 200 mN is dominated by 0.80 mm (> 99% confidence level) and 0.86 mm (> 95% confidence level) (Fig. 6d); while hardness at 200mN has peaks at 1.75 mm and 0.62–0.65 mm (> 95% confidence level) (Fig. 6e). Spectral analysis on modulus at 50 mN shows high-power peaks at 1.75 mm, 0.98 mm (> 99% confidence level), 1.08 mm and 0.59 mm (> 95% confidence level) (Fig. 6f); while hardness at 50mN has peaks at 1.06 mm (> 99% confidence level), 1.64 mm and 0.45 mm (> 95% confidence level) (Fig. 6g).

**Fig. 7:**
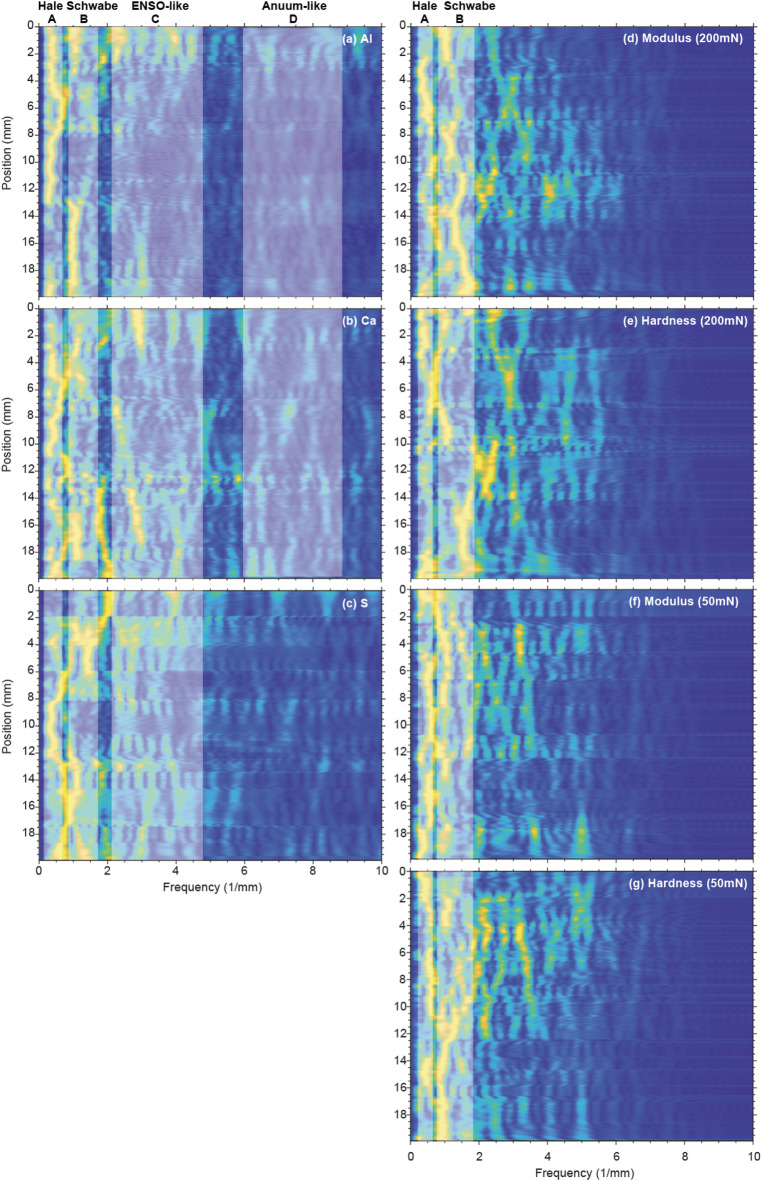
EFFT spectra of Al (**a**), Ca (**b**), S (**c**), nanomechanical properites at 200 mN (**d-e**) and 50 mN (**f-g**). Main cycles (A-D) are shadowed in gray.

**Fig. 8:**
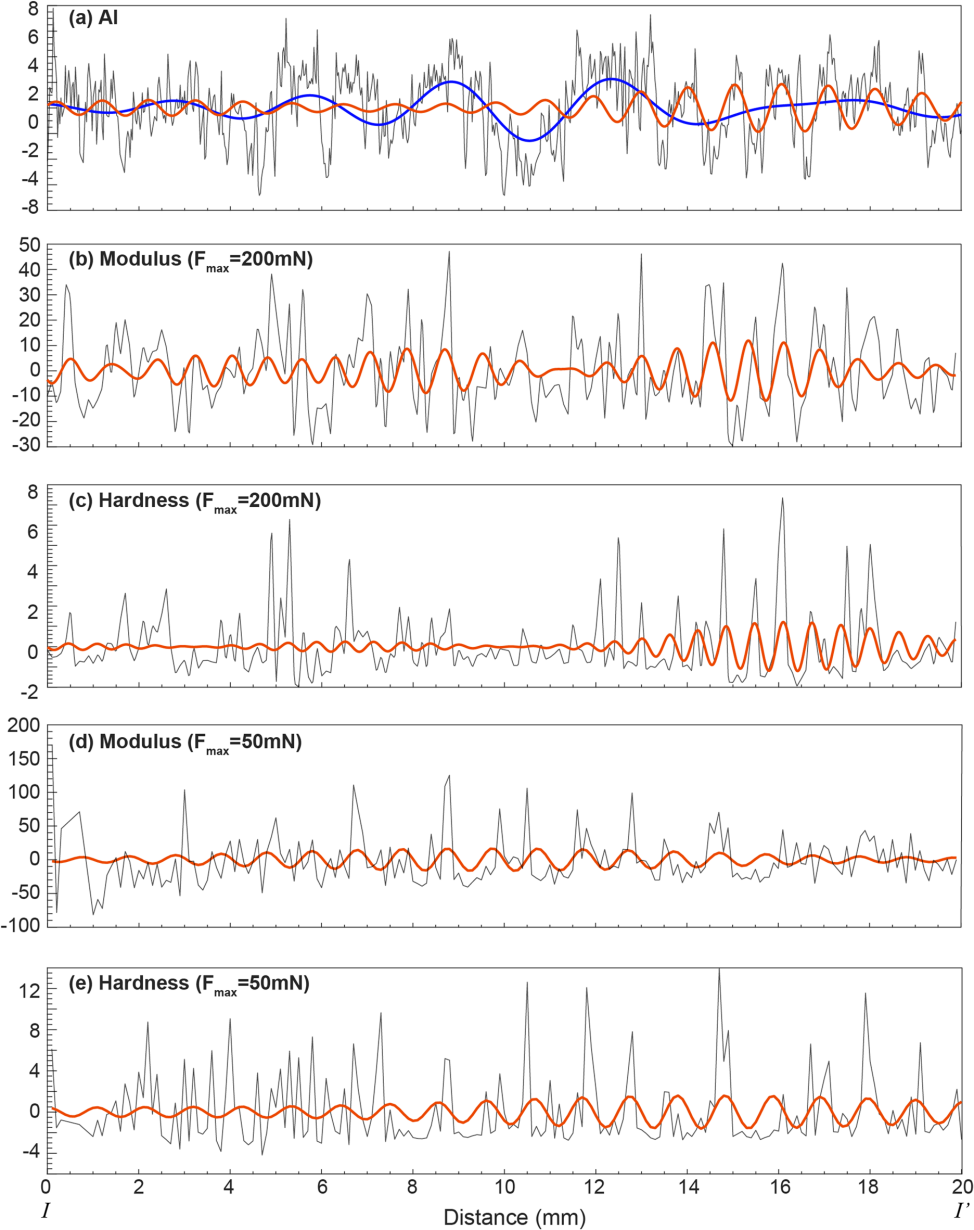
The detrended curves of Al (**a**), Young’s modulus and hardness at 200 mN and 50 mN (**b-e**) of line I-I’ in Fig. 2. The blue line in (**a**) represents ~ 3.57 mm cycle and was extracted from the detrended Al series with a bandpass of 0.28 ± 0.08 cycles/mm; the red lines represent 1.2–0.6 mm cycles and were extracted from the detrended series with a bandpass of 1.0 ± 0.05 cycles/mm in (** a**),1.2 ± 0.1 cycles/mm in (**b**), 1.6 ± 0.1 cycles/mm in (**c**), 1.0 ± 0.04 cycles/mm in (**d**), and 0.96 ± 0.04 cycles/mm in (**e**).

After comprehensively comparing peaks at > 95% confidence levels for geochemical and nanomechanical proxies, the major cycle bands of A (3.9–1.6 mm), B (1.2–0.6 mm), C (0.4–0.2 mm) and D (0.2–0.1 mm) are recognized for Al and Ca (Figs. 6a-b), while only cycles B and C for S (Fig. 6c). Modulus and hardness show major cycles of A and B (Figs. 6d-g). The EFFT spectrograms for detrended geochemical and nanomechanical series (Fig. 7) show consistent strong wavelength cycles with MTM power spectral analyses. The 1.2–0.6 mm cycles were extracted from Al and nanomechanical series (Figs. 8a-e), with a 3.57 mm cycle from Al series additionally (Fig. 8a).

### Wavelet analysis

#### Continuous wavelet transform

The wavelet scalograms for detrended geochemical and nanomechanical series are shown in Fig.S2. All figures share the same color bar. The color bar represents the value of wavelet transform coefficient, and a higher value means a stronger fluctuation. The statistically significant regions with over 95% confidence are designated by the bold black lines. The wavelet scalograms reveal that variations of Ca and Al are dominated by cycles A-D intermittently (Figs. S2 a and b), and S with cycles B and C (Figs. S2 c), while Young’s modulus and hardness mainly showing cycle B (Figs. S2 d-g).

### Wavelet coherence and cross wavelet transform

WTC and XWT plots between Al and Ca, Ca and S, Al and S are shown in Fig. S3. Based on phase relationship results, the negative correlations between Al and Ca, Al and S are mainly concentrated in the period of 2–6 mm scales throughout the whole section in WTC, and at location 3–17 mm in XWT, reflecting overall significant periodic variation characteristics (Figs. S3 a, b, e and f). Several cycles are also shown at approximately 0.125–1 mm sporadically (Figs. S3 a, b, e and f). By contrast, positive interaction is observed between Ca and S at period 2–6 mm throughout the whole section in WTC, and at period ~ 3 mm at location 6–10 mm in XWT (Figs. S3 b and c).

WTC and XWT between hardness and modulus at 50 mN and 200 mN are shown in Fig. S4. It is noticeable that hardness and Young’s modulus are positively related in the main cycles at 0.0625–0.5 mm, 2–4 mm, 5–6 mm in WTC plots, and 0.125–2 mm in XWT plots.

The relationship between elemental contents and nanomechanical properties at 200 mN and 50 mN on a time scale are shown in Figs. S5 and S6. It is found that compared to 50 mN, modulus and hardness at 200 mN had more significant correlations with all elements over a period of 4–6 mm on the whole according to WTC plots, with positive phase correlation with Al content (Figs. S5 a-d, Figs. S6 a-d), but negative one with Ca and S contents (Figs. S5 e-l, Figs. S6 e-l).

## Discussion

### Conversion from depth to time domain

Based on U-Pb zircon ages of three volcanic ash layers, the sedimentation rate of K_2_qn in GY3HC can be estimated as 8.2 cm/kyr using mean age values (Fig. 1b). Although the presence of interbedded sandstone may affect sedimentation rate (e.g., as illustrated by adjacent SK1s well, where the Quantou Formation, predominantly composed of sandstone, exhibits a rate of 16.33 cm/kyr^24^), it only constitutes a minor fraction (<2%) of K_2_qn in GY3HC. This influence is negligible, as the mudstone sedimentation rate is calculated to be 8.1 cm/kyr when assuming 2% sandstone content (Appendix).

**Fig. 9:**
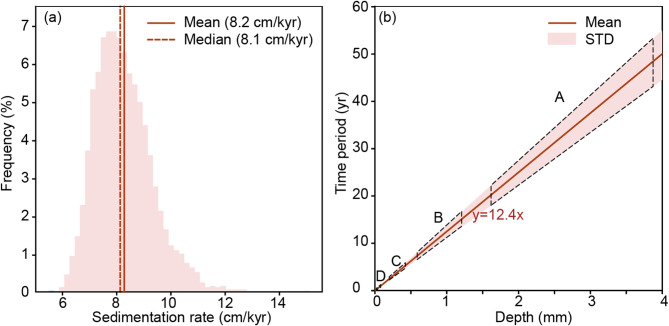
Histogram of sedimentation rate (**a**) and correlation between depth and time domains (**b**) modeled via Monte Carlo approach. Ranges for cycles A–D are outlined with black dash lines in (**b**).

To account for age uncertainties of ash layers, sedimentation rate is further estimated via Monte Carlo method by randomly selecting ages within errors. This yields a mean value of 8.2 cm/kyr (Fig. 9a) and sample age as ca. 90.53 Ma. A linear depth-time model incorporating standard deviation is established (Fig. 9b), enabling conversion of cycles A–D into temporal durations: 47.7 ± 5.2–20.3 ± 2.2 year, 15.1 ± 1.6–7.4 ± 0.8 year, 5.3 ± 0.6–2.5 ± 0.3 year and 2.0 ± 0.2–1.4 ± 0.1 year, respectively (Fig. 9b; Table 1).

**Table 1:** Conversion from depth to time domain for cycles A-D via Monte Carlo modeling.

Cycle	Depth domain (mm)		Time domain (yr)			
	Min	Max	Min		Max	
			Mean	Std	Mean	Std
A	1.6	3.9	20.3	2.2	47.7	5.2
B	0.6	1.2	7.4	0.8	15.1	1.6
C	0.2	0.4	2.5	0.3	5.3	0.6
D	0.1	0.2	1.4	0.1	2.0	0.2

*std = standard deviation.

### Decennial to annual-scale variations archived in elemental and Nanomechanical series

#### Quasi-seasonal characteristics

Based on the simplified varve model^42^, carbonate laminae might deposit in warm summers, as increased insolation inducing carbonate supersaturation and precipitation under high pH and temperature; while the dark laminae may have formed in winters, when phytoplankton are dying, precipitating and accumulating slowly with settling clay materials at the bottom of the lake, contributing to typical annual cycles in lacustrine sediments^7,11,43^.

In this study, the progradation of calcareous sediments indicates a relatively oxygenated, biologically favorable water environment in summer, which is also confirmed by increased manganese (Table S2). And the dark laminae with high abundance of terrestrial elements (Al, Si, K) are consistent with a eutrophic water environment with suboxic-anoxic condition in winter. The thickness of a light-dark lamina couplet being ~ 0.15 mm (Fig. S7) corresponds well with annual cycle D (0.1–0.2 mm), representing two seasonal depositional events within one year. This indicates the existence of a quasi-seasonal climate in the Songliao Basin during the Late Cretaceous.

#### ENSO-like climatic fluctuations

The high-frequency periodicity of cycle C identified in elemental series (5.3 ± 0.6–2.5 ± 0.3 year) (verified under microscope in Fig. S7) is comparable with present-day El Ni$\:\stackrel{\sim}{n}$o–Southern Oscillation (ENSO; 3–7 year), an internal mode of the coupled equatorial ocean-atmosphere system and could be self-sustaining or triggered by random noise^15^, being persistently active and robust since the Mesozoic^44,45^. ENSO-type signals are observed in marine sediments in Late Cretaceous, including Marca Shale in California^46^ and sediments in Arctic Ocean^47^. Meanwhile, ENSO-like variability has also been reported in Songliao Basin at 84.4 Ma with inter-annual periodicities of 2.2–2.7 year and 3.5–6.1 year^9^. Stratosphere-troposphere coupling played a prominent role in the transmission of Cretaceous equatorial climate forcing to middle, high and even polar latitudes. During late Cretaceous, the large land‐sea contrast and extensive mountain ranges in the northern hemisphere amplified tropospheric planetary waves. Specifically, due to the collision between Okhotomorsk Block and East Asia during 100–89 Ma, a coastal mountain range higher than 2 km was formed along the East Asian margin^48,49^, which likely enhanced planetary waves in mid-latitude areas in East Asia, e.g., Songliao Basin, thereby enhancing inland climatic responses to ENSO. Notably, the relatively low resolution of nanomechanical series restricts accurate identification of ENSO-like signals. However, if lowering the threshold with sampling frequency greater than twice the frequency of signal analyzed^41^, peaks with over 90% confidence level are recognizable for modulus and hardness in cycle C (Figs. 6d-g). This probably indicates ENSO-like climate fluctuation achieved in nonmechanical record, which should have been more reliable under higher resolution.

#### Solar activities

Decadal periods of A (47.7 ± 5.2–20.3 ± 2.2 year) and B (15.1 ± 1.6–7.4 ± 0.8 year) identified in elemental and nanomechanical series could be attributed to solar activities of Hale (~ 22 year) and Schwabe (~ 11 year; range of 8–17 year) cycles, as confirmed by band-pass filtering (Fig. 8). Schwabe cycle reflects changes in sunspot number and the latitudinal migration of solar eminences. And the Hale cycle results from solar magnetic field alternation between normal and reversed polarities during successive Schwabe cycles^50^. Millennial to decadal scale cyclicity related to solar activity had been suggested in Middle Cretaceous marine deposits in the Western Interior Seaway, North America during Cretaceous mid-Cenomanian event (MCE; 96.6–96.2 Ma; a “green-house” period)^51^. It is supposed that both MCE and oceanic anoxic event 2 (OAE2, ca. 94 Ma) occurred during 2.4 Myr eccentricity minima^52^, during which time solar forcing, especially Schwabe and Hale cycles, exerted strong impact on depositional environment^14,51^.

During K_2_qn sedimentation in the Songliao Basin, solar activities governed lamina types by controlling paleoclimate. During solar activity minima (low sunspot number), a weakened Sun’s magnetic field allowed increased galactic cosmic rays into atmosphere, which promoted low cloud cover with subsequent cool climate conducive to forming dark clastic laminae. In contrast, during solar activity maxima (high sunspot number), strengthened Sun’s magnetic field prevent galactic cosmic rays from entering atmosphere, leading to warm conditions for more light carbonate laminae^11^. Solar forcing ultimately imprinted its signal as rhythmic geochemical and mechanical variations with periods of (47.7 ± 5.2–20.3 ± 2.2 year and 15.1 ± 1.6–7.4 ± 0.8 year in the shale.

Notably, as no peaks exceeding 99% significance level for S, extraterrestrial solar activities and climatic fluctuations may not be the dominant factor influencing S variation (Fig. 6c). Intrinsic factors within the lacustrine system (e.g., water chemistry, lake stratification and biological cycle) could also produce laminae^53^. Sulfur in sediments, mainly in the form of pyrite (FeS_2_), is mediated by sulfate-reducing bacteria in anoxic water condition^54,55^. And the involvement of microbial metabolic process makes S cycle in lake system more complicated than others (e.g., Al and Ca in this study are redox-insensitive elements and are primarily modulated by solar forcing-controlled climate change), thereby weakening its response to solar forcing. This remind us that elemental proxies are differentially sensitive to internal and external forces.

#### Implications to crack propagation

Similar cycles achieved in elemental and nanomechanical series imply the elemental and/or mineralogical controls on nanomechanical properties of shale, while terrigenous detritus (represented by Al) contributes to much stiffer properties (Fig. 5). The minor discrepancy on decennial periodicities between elemental and nanomechanical variations may be originated from contact relationship and stacking mode among mineral grains.

**Fig. 10:**
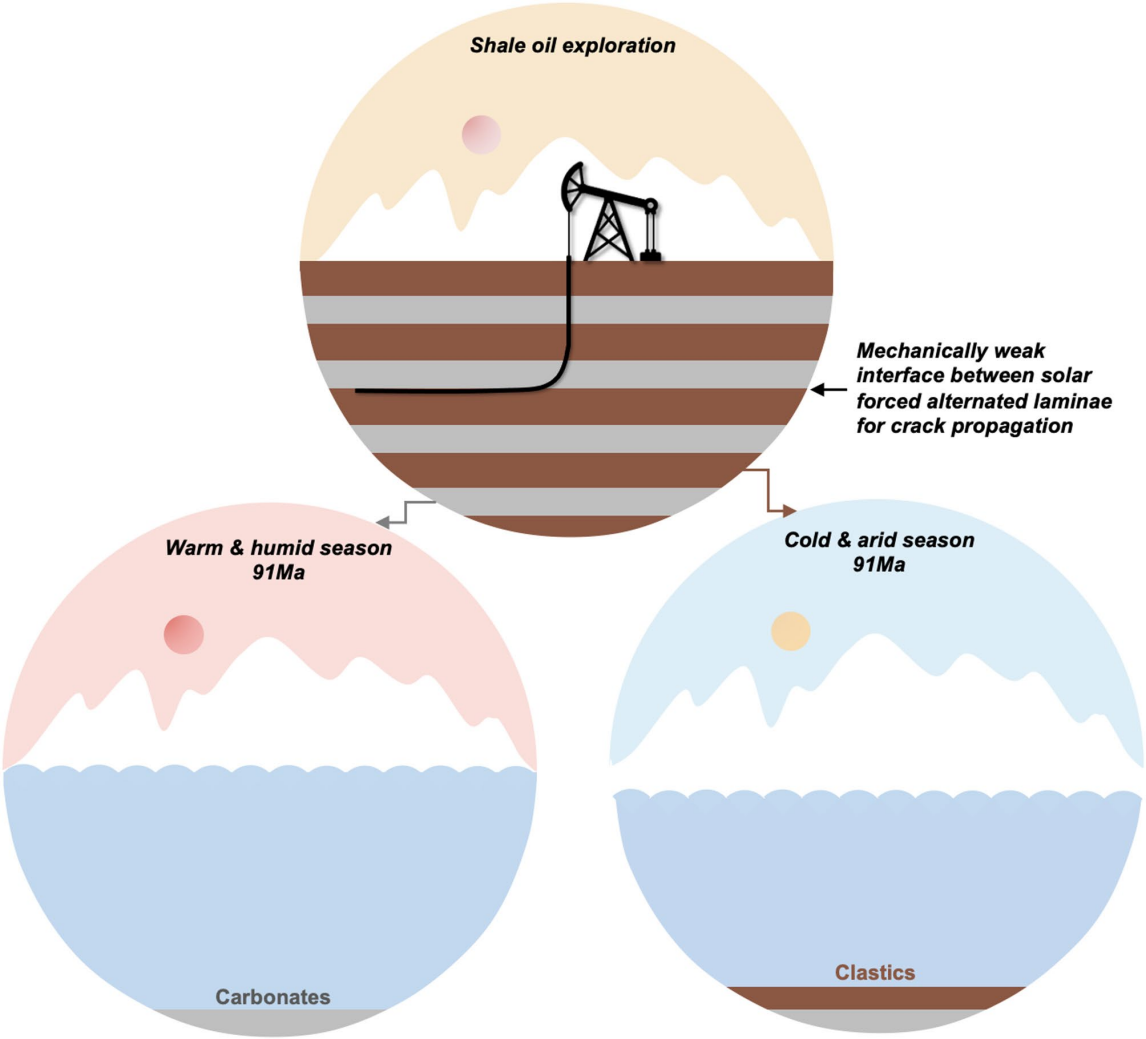
Simplified conceptual model for horizontal fracturing for shale oil/gas in lacustrine strata with carbonate-clastic layers. The mechanically weak interfaces between solar forced alternated laminae of soft carbonates precipitated in warm season and hard terrigenous clastic deposited in cold season, are susceptible to crack propagation.

The climatic shifts driven by solar cycles modulated sediment composition, e.g., terrigenous input and carbonate precipitation, which dictated rock mechanical properties and enabled the creation of weak interfaces that subsequently influencing fracture propagation. Minerals in lacustrine shales have extrabasinal (i.e., clay minerals, detrital quartz, feldspar, and terrestrial organic materials) and intrabasinal (i.e., calcite, dolomite, pyrite and bioclasts) sources, and the layered mixing of different sourced minerals with various mechanical properties is an important control on crack propagation. The dissimilar elastic properties between two contacting materials exert a significant influence on crack propagation at an interface^56^. The mechanically weak interface between alternated laminae of biogenically derived soft carbonates and brittle terrigenous detritus in this shale sample most likely renders it susceptible to crack propagation, contributing to complex fracture system during horizontal hydraulic-fracture treatment for shale oil exploration in Songliao Basin (Fig. 10). These high-density bedding fractures could lead to a complex fracture morphology around the wellbore, while retaining a large amount of fracturing liquid and restricting fracture expansion to the targeted layers in distance^57^, being a main challenge in effective shale oil exploration^58^. Then, the idea of “controlling near-wellbore fractures and maximizing main fracture extension” has been advocated^4^.

## Conclusion

Cyclicity analyses (including spectral and wavelet analyses) are conducted on high-resolution records of elemental and nanomechanical variations in a lacustrine laminated shale-carbonate couplet from Upper Cretaceous Qingshankou Formation in Songliao Basin, NE China ca. 90.53 Ma. Main ideas include:

1. Representative elements of Al, Ca and S reveal periodicities of 47.7 ± 5.2–20.3 ± 2.2 year and 15.1 ± 1.6–7.4 ± 0.8 year, corresponding to Hale and Schwabe solar cycles, respectively, while a 5.3 ± 0.6–2.5 ± 0.3 year cycle probably reflects ENSO-like climatic fluctuations.
2. The response of S to solar forcing and ENSO-like event is weakened, due to a complicated S cycle in lake water as influenced by intrinsic factors (e.g., redox condition and microbial activities), while Al and Ca are redox-insensitive and primarily governed by climate changes modulated by solar activities.
3. Solar cycles, particularly the Schwabe, are recognized in nanomechanical properties (Young’s modulus and hardness), suggesting that elemental and/or mineralogical composition exerts significant control over the nanomechanical behavior of shale rocks.
4. The mechanically weak interfaces between carbonate and detrital laminae in shales modulated by solar forcing resulted in high-density bedding fractures susceptible to crack propagation, which severely affected horizontal hydraulic fracturing design during shale oil exploitation.

This study bridges science and engineering with an integrated approach. Climatic changes driven by solar activities could alter aquatic environment, thereby leading to laminated sedimentary composition and mechanical properties. This layered structure with weak interfaces consequently governed the effectiveness of hydraulic fracturing. However, This paradigm requires further validation through engineering applications.

## Supplementary Information

Below is the link to the electronic supplementary material.

Supplementary Material 1

## Data Availability

Data is available for request to corresponding author Shuichang Zhang (sczhang@petrochina.com.cn) or Kunning Cui (cuikunning@petrochina.com.cn).
