# Supplementary material for: Solar forcing on elemental and nanomechanical variations in Late Cretaceous lacustrine deposits
Source: Sci Rep. 2025 Dec 21;15:44256. doi: 10.1038/s41598-025-27521-9 (PMC12722320; doi:10.1038/s41598-025-27521-9)
Supplement: Supplementary file 1 — Supplementary Material 1 [file 41598_2025_27521_MOESM1_ESM.docx]

Appendix:

To eliminate interference from sandstone, the sedimentation rate for pure mudstone can be obtained as:

$\frac{1}{R}=\frac{P}{R_{s}}+\frac{1-P}{R_{m}}$ (1)

where *R* is general sedimentation rate derived from ash layer ages as 8.2 cm/kyr, *R_s_* is sedimentation rate for the sandstone as 16.33 cm/kyr, *R_m_* is sedimentation rate for pure mudstone, *P* is the proportion of sandstone in sediments. The relationship between sandstone proportion and sedimentation rate is shown in Fig. A1.


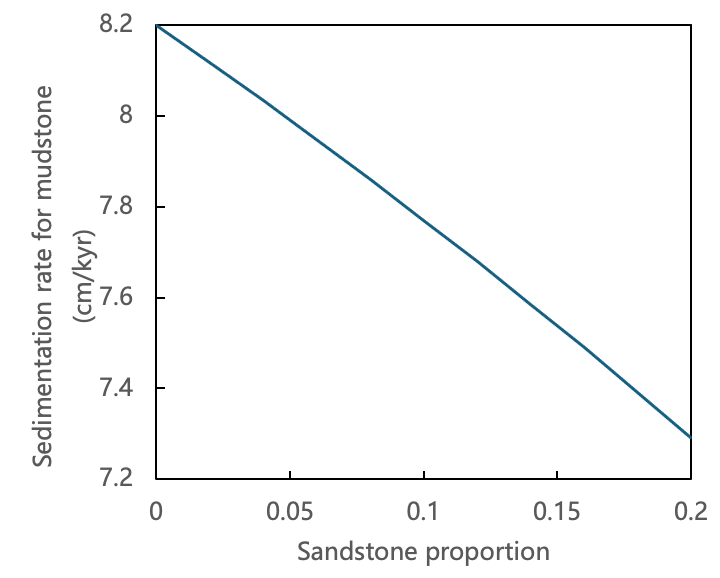


Fig. A1. Relationship between sandstone proportion and sedimentation rate in K_2_qn in GY3HC.


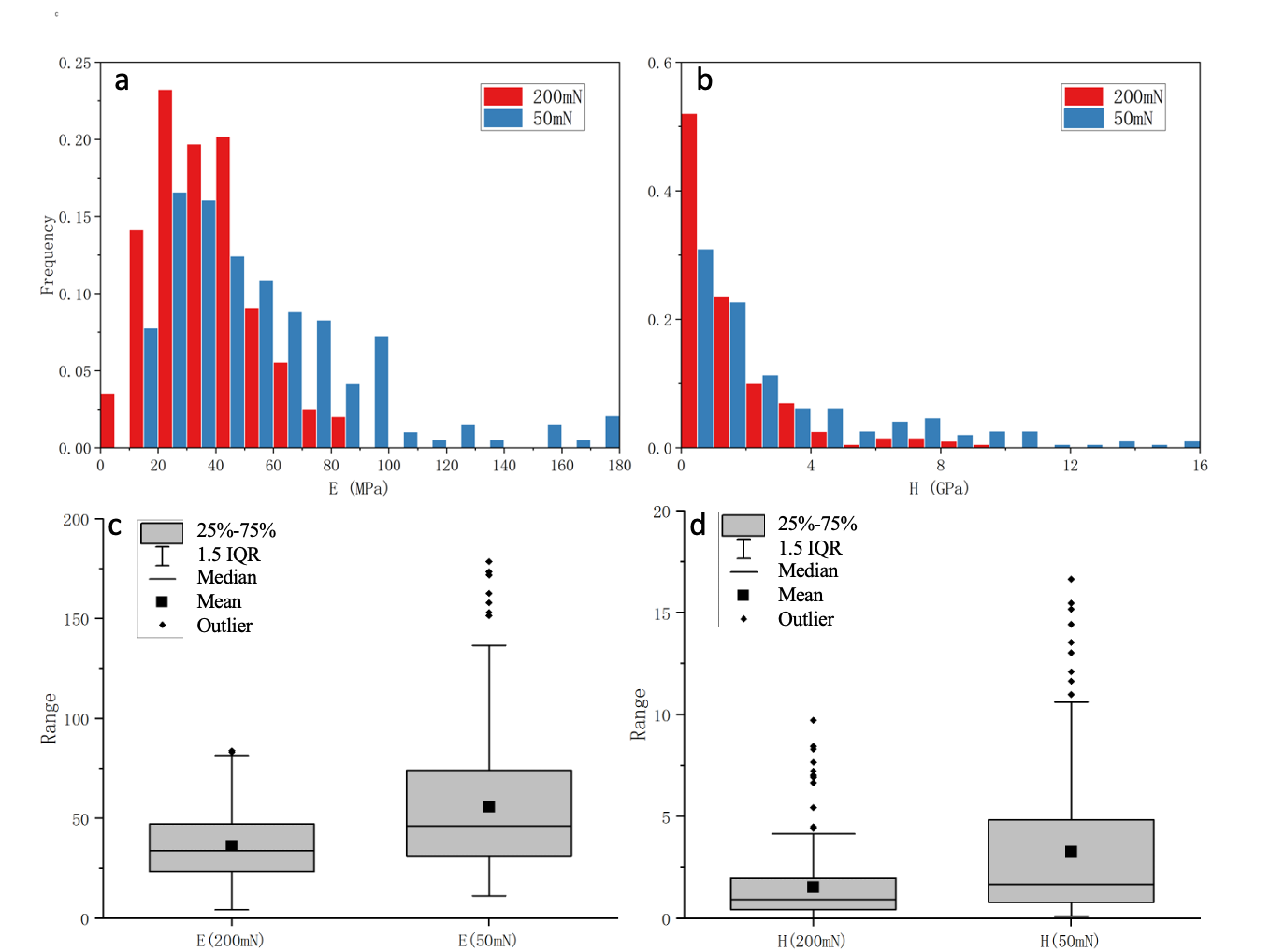


Fig. S1. Histogram (a and b) and boxplot (c and d) of modulus (E) and hardness (H) under 200mN and 50 mN.


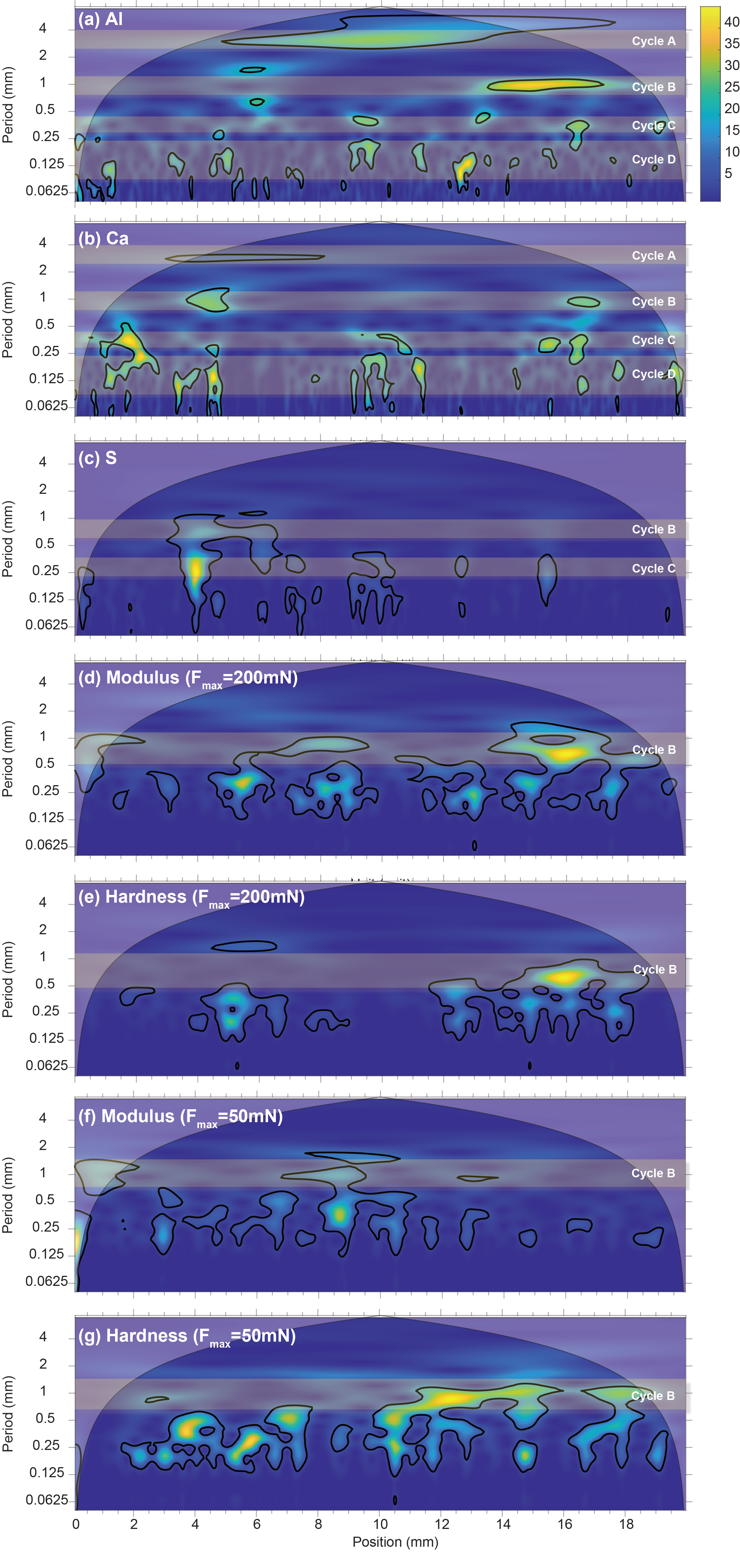


Fig. S2. The continuous wavelet power spectra of detrended Ca (a), Al (b), S (c), Young’s modulus and hardness at 200 mN (d and e) and 50 mN (f and g). All images share the same color bar, which represents the value of wavelet transform coefficient. The thick contour encloses regions of greater than 95% confidence for a red-noise process. Cross-hatched regions on either end indicate the “cone of influence,” where edge effects become important. Major cycles are highlighted in yellow.


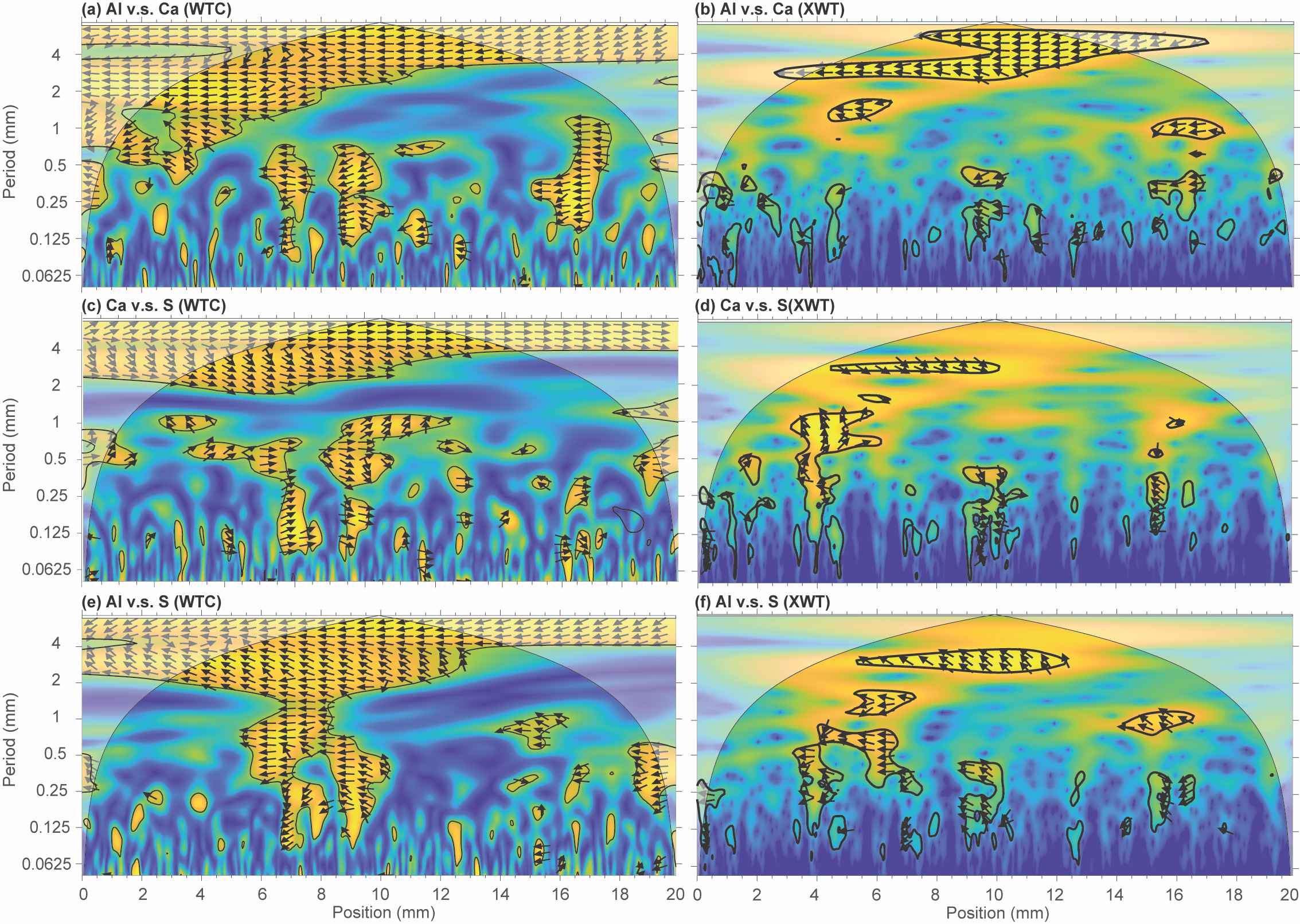


Fig. S3. Wavelet coherent plots (WTC) and cross wavelet transform plots (XWT) between Al and Ca (a and b), Ca and S (c and d), Al and S (e and f). All images share the same color bar. The thick contour encloses regions of greater than 95% confidence for a red-noise process. Cross-hatched regions on either end indicate the “cone of influence” where edge effects become important. Arrows denote relative phase difference: the arrows pointing to right indicate that the two series are in the same phase with a positive correlation; the arrows pointing to left indicate an inverse phase with a negative correlation; the downward arrows indicate that the former series is 90° ahead of the latter change; and the upward arrows indicate that the former series is 90° lagging the latter change.


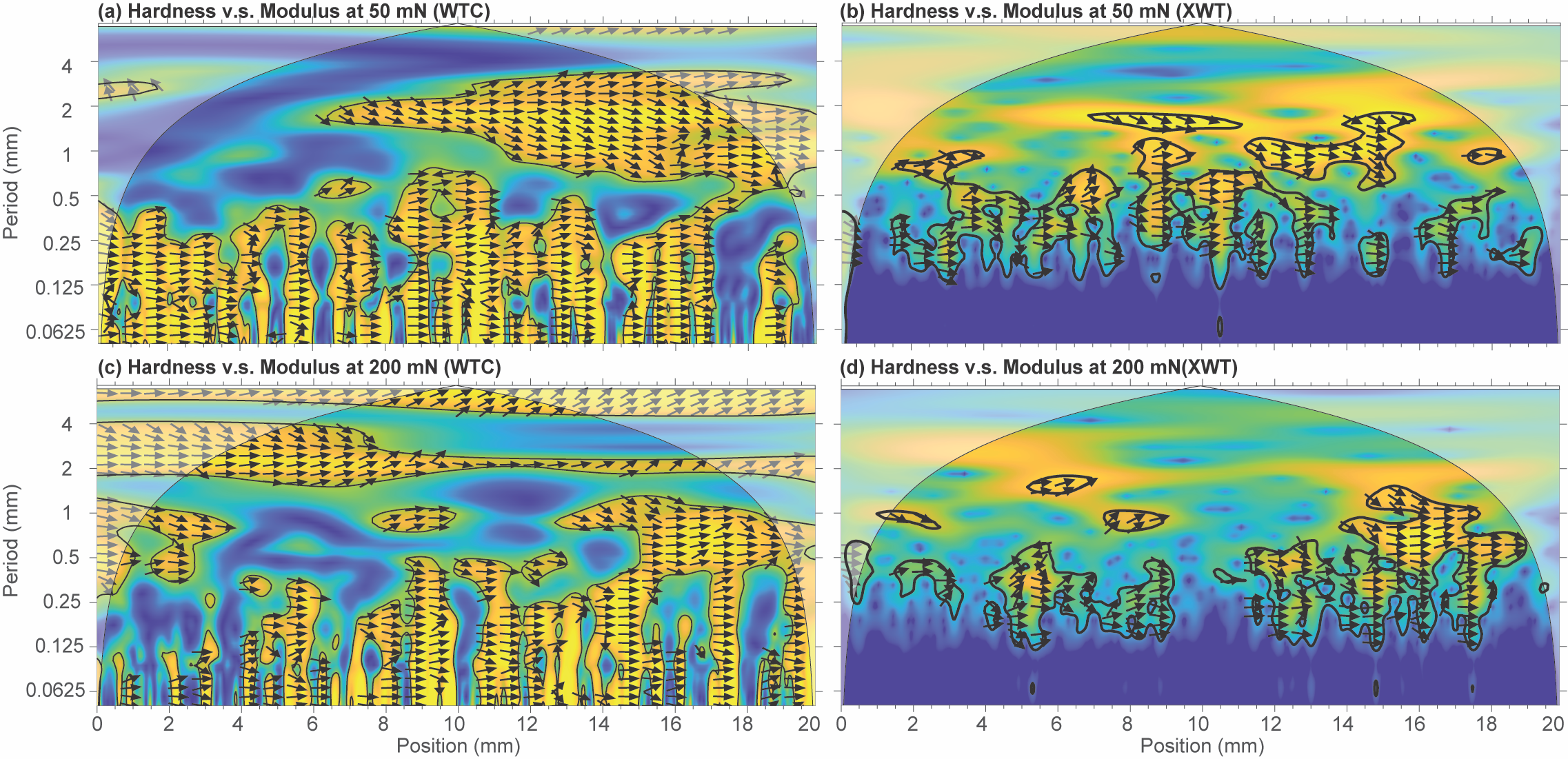


Fig. S4. Wavelet coherent plots (WTC) and cross wavelet transform plots (XWT) between hardness and modulus obtained at 50mN (a and b) and 200mN (c and d). All images share the same color bar. The thick contour encloses regions of greater than 95% confidence for a red-noise process. Cross-hatched regions on either end indicate the “cone of influence” where edge effects become important. Arrows denote relative phase difference: the arrows pointing to right indicate that the two series are in the same phase with a positive correlation; the arrows pointing to left indicate an inverse phase with a negative correlation; the downward arrows indicate that the former series is 90° ahead of the latter change; and the upward arrows indicate that the former series is 90° lagging the latter change.


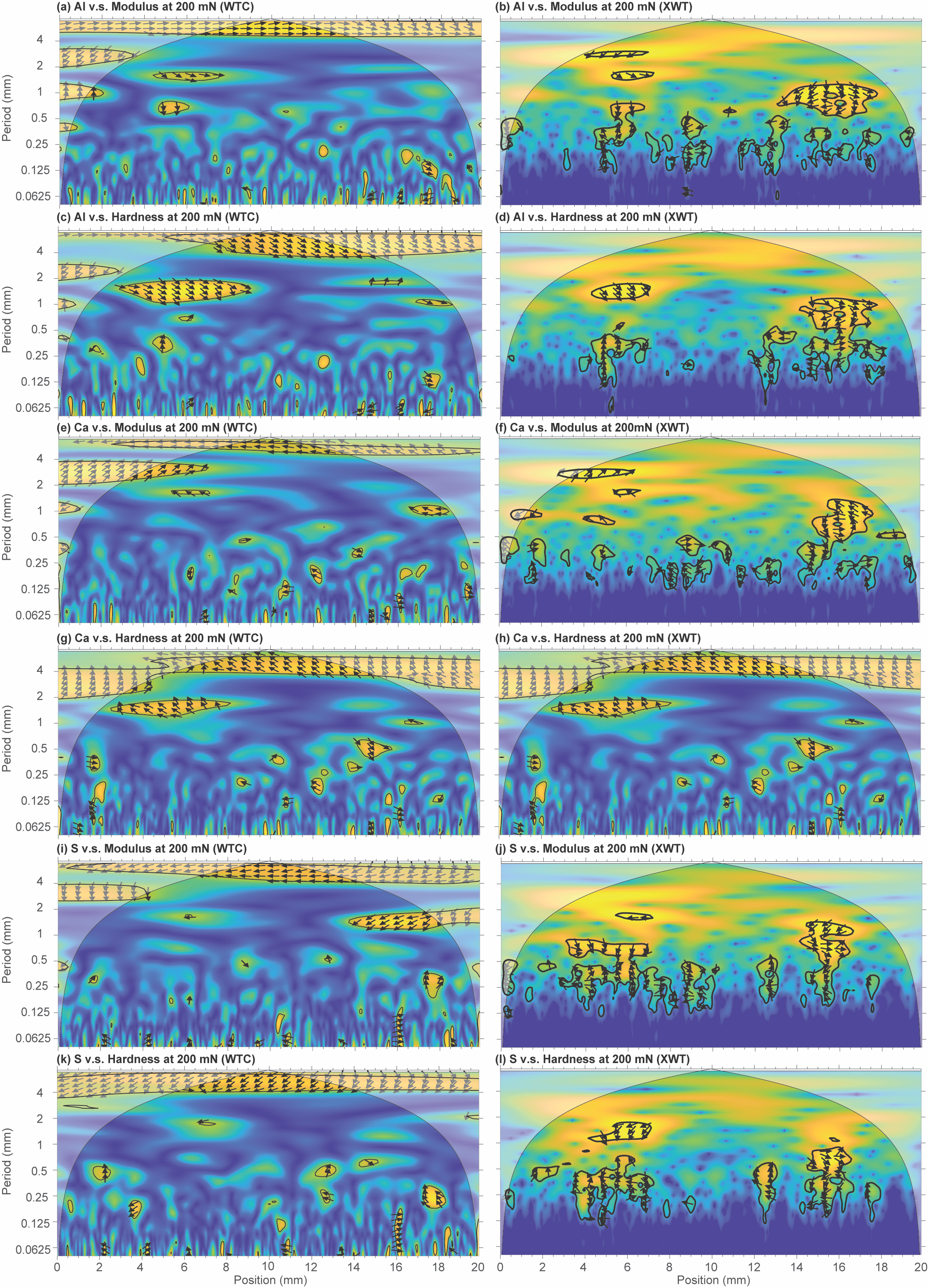


Fig. S5. Wavelet coherent plots (WTC) and cross wavelet transform plots (XWT) between elemental contents (Al, Ca and S) and nanomechanical properties (Young’s modulus and Hardness) obtained at 200mN. All images share the same color bar. The thick contour encloses regions of greater than 95% confidence for a red-noise process. Cross-hatched regions on either end indicate the “cone of influence” where edge effects become important. Arrows denote relative phase difference: the arrows pointing to right indicate that the two series are in the same phase with a positive correlation; the arrows pointing to left indicate an inverse phase with a negative correlation; the downward arrows indicate that the former series is 90° ahead of the latter change; and the upward arrows indicate that the former series is 90° lagging the latter change.


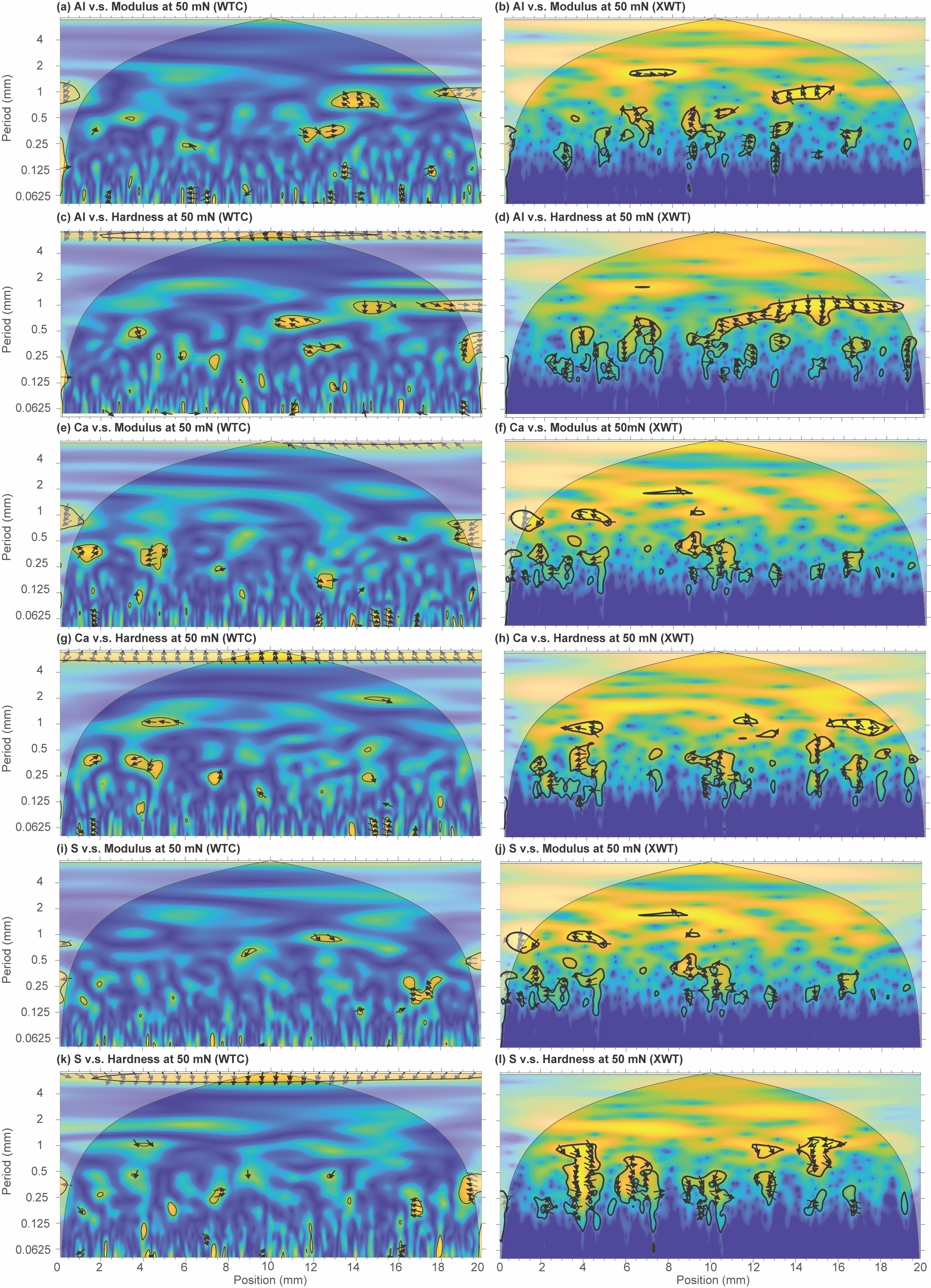


Fig. S6. Wavelet coherent plots (WTC) and cross wavelet transform plots (XWT) between elemental contents (Al, Ca and S) and nanomechanical properties (Young’s modulus and Hardness) obtained at 50mN. All images share the same color bar. The thick contour encloses regions of greater than 95% confidence for a red-noise process. Cross-hatched regions on either end indicate the “cone of influence” where edge effects become important. Arrows denote relative phase difference: the arrows pointing to right indicate that the two series are in the same phase with a positive correlation; the arrows pointing to left indicate an inverse phase with a negative correlation; the downward arrows indicate that the former series is 90° ahead of the latter change; and the upward arrows indicate that the former series is 90° lagging the latter change.


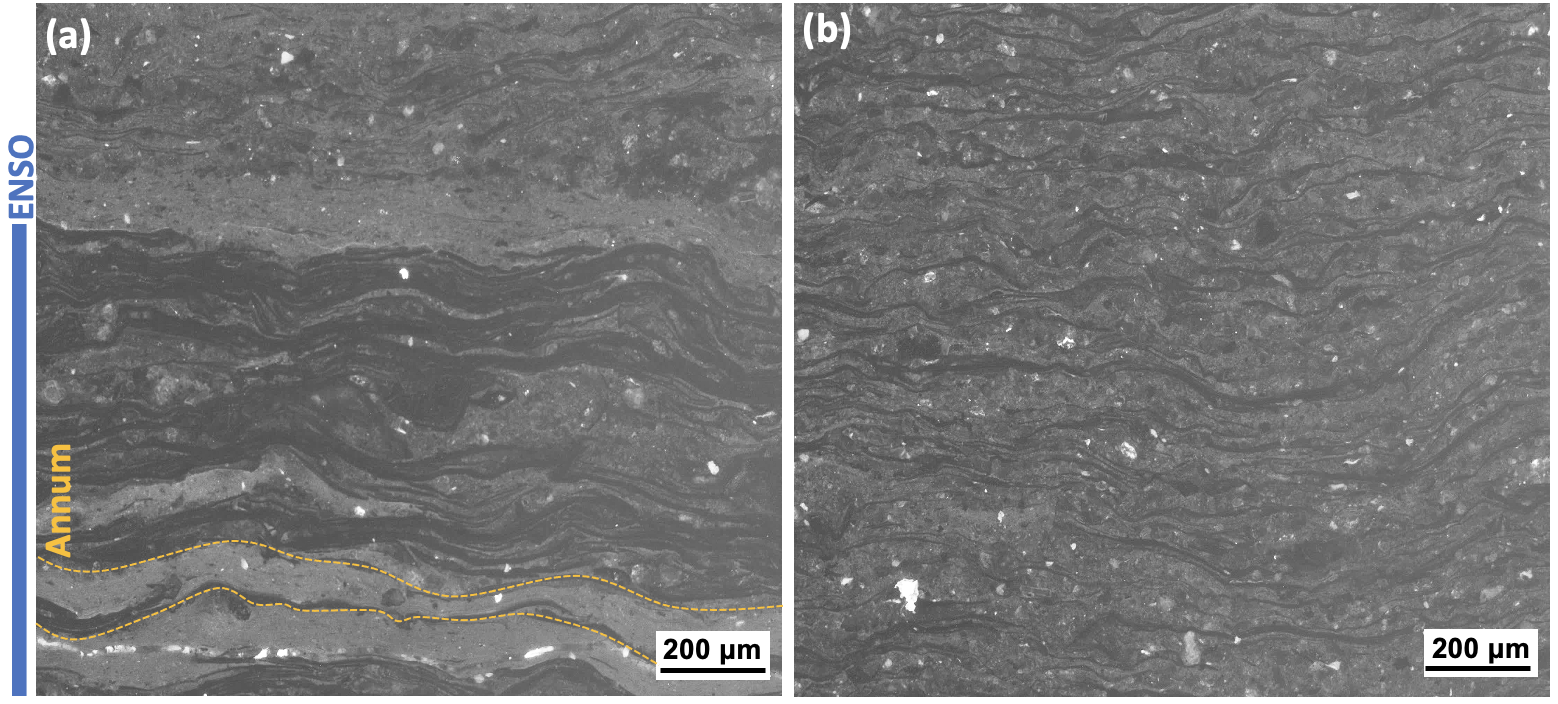


Fig. S7. Microscopic photos of shale with light and dark laminae distributed unevenly (a) and evenly (b). A couplet consisting of light and dark laminae is outlined as possible annual cycle. And the possible scale of ENSO event is marked.

Table S1. Principal component loadings on the major elements shown in Fig. 2.

| Element | PC1 | PC2 | PC3 | Group |
| --- | --- | --- | --- | --- |
| Si | -0.33394 | -0.02022 | -0.10369 | Terrigenous input |
| Al | -0.42425 | 0.19483 | 0.24707 |  |
| Mg | -0.17192 | 0.35104 | 0.78243 |  |
| K | -0.40934 | 0.06974 | 0.05746 |  |
| Ca | 0.38186 | -0.29721 | 0.37496 | Carbonate productivity |
| Mn | 0.3717 | -0.2924 | 0.39294 |  |
| Fe | 0.33217 | 0.58973 | -0.06279 | Pyrite signal |
| S | 0.34214 | 0.5585 | -0.11734 |  |

Table S2. Correlations between every two elements (Cells with values higher than 0.5 and lower than -0.5 are shaded with orange and green, respectively).

| Elements | Si | Al | Mg | K | Ca | Mn | Fe | S |
| --- | --- | --- | --- | --- | --- | --- | --- | --- |
| Si | 1 | 0.47941 | 0.17269 | 0.46403 | -0.48641 | -0.43058 | -0.39616 | -0.41657 |
| Al | 0.47941 | 1 | 0.4876 | 0.8369 | -0.61573 | -0.58868 | -0.41161 | -0.43211 |
| Mg | 0.17269 | 0.4876 | 1 | 0.2333 | -0.19717 | -0.18196 | -0.00765 | -0.09186 |
| K | 0.46403 | 0.8369 | 0.2333 | 1 | -0.55987 | -0.54793 | -0.46775 | -0.45443 |
| Ca | -0.48641 | -0.61573 | -0.19717 | -0.55987 | 1 | 0.84388 | 0.26858 | 0.30097 |
| Mn | -0.43058 | -0.58868 | -0.18196 | -0.54793 | 0.84388 | 1 | 0.27569 | 0.28627 |
| Fe | -0.39616 | -0.41161 | -0.00765 | -0.46775 | 0.26858 | 0.27569 | 1 | 0.97232 |
| S | -0.41657 | -0.43211 | -0.09186 | -0.45443 | 0.30097 | 0.28627 | 0.97232 | 1 |
